# Activating words without language: beta and theta oscillations reflect lexical access and control processes during verbal and non-verbal object recognition tasks

**DOI:** 10.1093/cercor/bhac499

**Published:** 2023-02-01

**Authors:** Francesca M Branzi, Clara D Martin, Emmanuel Biau

**Affiliations:** Department of Psychological Sciences, Institute of Population Health, University of Liverpool, Liverpool L69 7ZA, UK; BCBL. Basque Center on Cognition, Brain and Language, Paseo Mikeletegi 69, San Sebastian 20009, Spain; IKERBASQUE, Basque Foundation for Science, Maria Diaz de Haro 3, Bilbao 48013, Spain; Department of Psychological Sciences, Institute of Population Health, University of Liverpool, Liverpool L69 7ZA, UK

**Keywords:** cognate, beta, lexical access, EEG, semantic

## Abstract

The intention to name an object modulates neural responses during object recognition tasks. However, the nature of this modulation is still unclear. We established whether a core operation in language, i.e. lexical access, can be observed even when the task does not require language (size-judgment task), and whether response selection in verbal versus non-verbal semantic tasks relies on similar neuronal processes. We measured and compared neuronal oscillatory activities and behavioral responses to the same set of pictures of meaningful objects, while the type of task participants had to perform (picture-naming versus size-judgment) and the type of stimuli to measure lexical access (cognate versus non-cognate) were manipulated. Despite activation of words was facilitated when the task required explicit word-retrieval (picture-naming task), lexical access occurred even without the intention to name the object (non-verbal size-judgment task). Activation of words and response selection were accompanied by beta (25–35 Hz) desynchronization and theta (3–7 Hz) synchronization, respectively. These effects were observed in both picture-naming and size-judgment tasks, suggesting that words became activated via similar mechanisms, irrespective of whether the task involves language explicitly. This finding has important implications to understand the link between core linguistic operations and performance in verbal and non-verbal semantic tasks.

## Introduction

Imagine you are at home getting ready to go out. Someone asks you which type of shoes you intend to wear. Your intention to speak will likely activate lexical and phonological representations corresponding to “boots,” because retrieving these representations is necessary to respond to your interlocutor (Strijkers et al. 2012; Branzi et al. 2021). However, what happens to those lexical representations if now the task requires to “tidy up” and decide whether these boots fit in a shoe box? Would the word representations corresponding to “boots” be activated, even without mandatory and explicit word-retrieval?

Past research has shown that the intention to name an object modulates the neural network and the event-related potential (ERP) responses during object recognition tasks (Strijkers et al. 2012; Branzi et al. 2021). However, the nature of this modulation is still unclear. The current literature has not yet established whether the intention to name an object facilitates activation of lexical/phonological representations (Strijkers et al. 2012), or whether it is a necessary requisite for observing lexicalization processes (Meyer et al. 1998; Jescheniak et al. 2002; Bles and Jansma 2008; Branzi et al. 2021).

Here, we investigate whether lexical access takes place even when there is no intention to name (i.e. overtly using language), and whether the neural processes supporting lexical access are quantitatively and/or qualitatively similar to when explicit word-retrieval is intended. Addressing these questions not only is crucial to characterize the neural and cognitive basis of lexical access, a core operation in language, but also to determine whether response selection during semantic tasks requiring verbal versus non-verbal responses rely on similar neuro-computations (Fedorenko and Shain 2021; Ivanova et al. 2021).

Neural oscillations are particularly suitable to address these questions as they reveal the precise timing of the neural dynamics reflecting spreading activation during lexical access. Accordingly, neuronal oscillation patterns already allowed to establish a link between cognitive and neurophysiological computations in various cognitive domains (Siegel et al. 2012; Friederici and Singer 2015), including language (Meyer 2018; Piai and Zheng 2019).

For instance, alpha-beta and theta frequency bands have been often associated to word-retrieval during picture-naming and verb generation tasks ( Ojemann et al. 1989; Piai et al. 2014b; Piai et al. 2015; Piai et al. 2016; Jafarpour et al. 2017; Piai et al. 2017; Piai et al. 2018). Yet, alpha-beta and theta oscillations have been related to different cognitive operations during word-retrieval. On the one hand, alpha-beta power desynchronization (or alpha-beta power decrease) has been related to activation of information within the lexical-semantic system (Piai et al. 2015; Roos and Piai 2020). On the other hand, similarly to the domain of action monitoring (Trujillo and Allen 2007; Cavanagh et al. 2009; Cohen 2011), theta power increases have been associated to cognitive control demands—and especially monitoring control—during the retrieval of lexical representations in language tasks (Piai and Zheng 2019; Geng et al. 2022).

In the present electroencephalogram (EEG) study, we tested a group of healthy participants and compared their oscillatory activity focusing on alpha-beta and theta frequencies during two different tasks—a picture-naming task and a size-judgment task. Importantly, both picture-naming and size-judgment tasks relied on similar picture processing operations (extraction of visual features, visual-semantic processing for object recognition, response selection) but only one, the picture-naming task, required explicit retrieval of object names. In contrast, the size-judgment task required participants to make a size-judgment providing a manual response to indicate whether an object was “bigger” or “smaller” than a shoebox, but no explicit retrieval of the object name.

Since the retrieval of semantic information has been linked to both alpha-beta desynchronization and theta synchronization irrespective of the intention to speak (e.g. in semantic-based episodic memory tasks; see Piai and Zheng 2019), we expected that visual object processing would induce an overall significant decrease in alpha-beta power (i.e. desynchronization) as well as an increase in theta power (i.e. synchronization) relative to the time-window preceding the onset of the object presentation, in both picture-naming and size-judgment tasks. Nevertheless, the main goal of the present study was to examine whether the intention to name an object modulated the access to lexical information specifically. Therefore, we manipulated the “cognate status” of the stimuli in the two tasks (picture-naming and size-judgment).

The cognate status of a word is determined by the extent to which it shares orthographic and phonological features with its translation equivalent in another language. Cognates are translation words that have similar orthographic–phonological forms in two languages (e.g. *tomato*—English, *tomate*—Spanish). By contrast, non-cognates are translation equivalents that share only their meaning (e.g. *apple*—English, *manzana*—Spanish). Typically, in bilingual speakers, behavioral and neural differences between non-cognate and cognate conditions are observed during picture-naming and indicate lexical/phonological activity (the “cognate effect,” see Costa et al. 2000; De Bleser et al. 2003; Costa et al. 2005; Christoffels et al. 2007; Strijkers et al. 2010; Branzi et al. 2021). Therefore, as in previous studies, here we tested bilingual participants, and we employed the behavioral and neural cognate effect as a proxy for lexical/phonological activity (Strijkers et al. 2010; Branzi et al. 2021) and examined whether this effect varied as a function of the intention to name an object (Branzi et al. 2021). In fact, since the cognate status of a word is defined by formal overlap and is not correlated with any perceptual or conceptual variable (Costa et al. 2000; De Bleser et al. 2003; Costa et al. 2005; Christoffels et al. 2007; Strijkers et al. 2010; Palomar-Garcia et al. 2015), any behavioral or neural difference between non-cognate and cognate processing would reflect a purely lexical/phonological effect.

If a cognate effect were found in both tasks, it would indicate that visual object processing induces automatic activation of lexical/phonological representations, independently of the intention to speak (“spreading activation,” see Dell 1986; Caramazza 1997; Strijkers et al. 2012; see also evidence from the picture-word interference studies, i.e. Schriefers et al. 1990; Jescheniak and Schriefers 2001; de Zubicaray et al. 2002; picture-picture interference studies, Tipper and Driver 1988; Bles and Jansma 2008; but see other models which do not assume spreading activation in all circumstances, e.g. Levelt 1989; Levelt et al. 1999). If so, enhanced behavioral performance, i.e. faster reaction times and increased accuracy measures for cognates as compared to non-cognates should be observed in both picture-naming and size-judgment tasks (Strijkers et al. 2010; Branzi et al. 2020; Branzi et al. 2021).

Despite the evidence is still scarce, the current literature indicates that retrieving lexical information from memory is associated with power decreases in the alpha-beta band, similarly to the episodic-memory domain (Piai and Zheng 2019). Since lexical retrieval depends on the activation level of the target lexical representations (Dell 1986; Levelt et al. 1999; Finkbeiner et al. 2006), a greater desynchronization of neural alpha-beta oscillations should be observed for cognates as compared to non-cognates. Note that if this neural cognate effect were found in both tasks, it would indicate that lexical access during object naming and object-size categorization reflects the same spreading activation mechanism. Still, the neural cognate effect might be observed in an earlier time-window for the picture-naming task as compared to the size-judgment task (Strijkers et al. 2012), which would indicate similar processes across tasks, but faster when explicit word-retrieval is required.

Furthermore, in the present study, the intention to name an object (picture-naming task) may induce faster dual-language activation (Strijkers et al. 2012), which should affect cognates only. In fact, cognate, but not non-cognate words, receive activation from both the target (e.g. *tomato* in English) and non-target (translation equivalent, e.g. *tomate* in Spanish) languages. This simultaneous activation might increase conflict between target and non-target translation equivalents that share lexical/phonological representations (cognate) and therefore the need for monitoring control (Li and Gollan 2018). Therefore, if theta power reflects monitoring control processes during response selection (Cavanagh and Frank 2014; Cohen 2014; Geng et al. 2022), this effect should be stronger in the picture-naming task as compared to the size-judgment task for cognate stimuli only, in accord with the view that theta oscillations synchronize when conflict between representations increases (Cavanagh and Frank 2014; Cohen 2014; Geng et al. 2022).

Finally, we investigated whether during picture-object processing the neural processes reflecting activation of lexical knowledge, hypothetically indexed by alpha-beta activity, interacted with monitoring control processes, hypothetically supported by theta activity. We evaluated the functional interaction between the theta phase and the alpha-beta amplitude during the two tasks, using cross-frequency coupling analysis, which allows at establishing whether the two different oscillatory processes under examination (alpha-beta and theta oscillations) exhibit “yoked” time-courses—i.e. constitute highly interacting rather than independent oscillatory processes during the two tasks.

We hypothesized that the intention to name an object would modulate interactions between two different oscillations reflecting distinct cognitive processes during picture-object processing. In other words, we hypothesized that similar to the expected theta power effects, the functional interaction between theta and alpha-beta oscillatory responses would be modulated by increased control demands during response selection. Thus, in accordance with working memory research studies that have shown an increase in beta-theta coupling proportional to an increase in control demands (Daume et al. 2017a; Daume et al. 2017b), increased theta-beta coupling should be observed in the picture-naming task as compared to the size-judgment task for cognate stimuli only, i.e. those conditions requiring increased monitoring control demands.

**Fig. 1:**
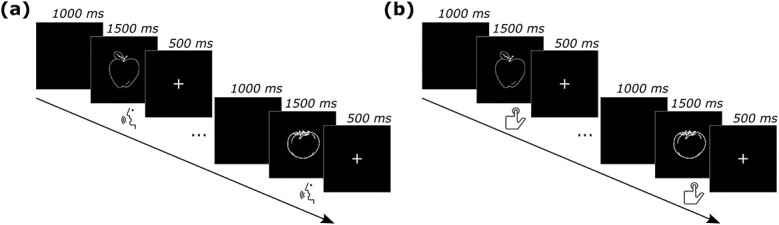
Experimental paradigm. Schematic depiction of trial structure and timing for both (a) the picture-naming task and (b) the size-judgment task. During the same experimental session, participants performed a block of picture-naming and a block of size-judgment task. The order of the tasks was counterbalanced across participants. In each task, participants were presented with 32 pictures of objects referring to cognate stimuli (e.g. *tomàquet*—Catalan, *tomate*—Spanish) and 32 pictures of objects referring to non-cognate stimuli (e.g. *poma*—Catalan, *manzana*—Spanish). For each participant, the pictures of objects were not repeated across the two tasks.

## Materials and methods

### Participants

Twenty early and high-proficient Catalan/Spanish bilinguals took part in the experiment and received monetary remuneration for their participation (12 females; mean age: 21.3 years, standard deviation (SD) = 1.9; for further details, see Branzi et al. 2014). Half of the participants were dominant in Spanish, whereas others were dominant in Catalan. However, all participants were early bilinguals, equally proficient in both languages (self-assessed proficiency scores are reported in Branzi et al. 2014). All participants were right-handed and had a normal or corrected-to-normal vision. The data set of two participants was excluded from the analyses due to excessive movement artifact contamination. The analyses were applied to the remaining eighteen participants. The study was approved by the local ethics committee. Written informed consent was obtained from all participants.

### Stimuli

One hundred and twenty-eight line-drawings of objects were selected from different databases (Snodgrass and Vanderwart 1980; Szekely et al. 2004). The pictures represented objects belonging to a wide range of semantic categories (e.g. animals, body parts, buildings, furniture). In both the picture-naming and size-judgment tasks, the objects depicted in the pictures could refer to either cognate or non-cognate words. The cognate status of the corresponding names was controlled to present 50% of the items as Spanish/Catalan cognates and the other 50% as non-cognates. The mean lexical frequency (LEXESP; Sebastián-Gallés et al. 2000) of the picture names was balanced between cognates and non-cognates (non-cognates: 1.03, SD = 0.6; cognates: 1.14, SD = 0.6; *t* (126) = −1.075, *P* = 0.284). Pictures were grouped together in order to create four experimental lists, which were then randomized across participants. Even if each participant was not presented with the very same pictures during the picture-naming and size-judgment tasks (see below), the very same stimuli and lists were employed for the two tasks across participants.

### Experimental procedure: picture-naming and size-judgment tasks

After having filled in the informed written consent and a language use/proficiency questionnaire, participants were tested individually in a soundproof room. Written instructions were presented in their native language (L1). Participants performed a picture-naming task and a size-judgment task in their L1^1^ during a single session. In both picture-naming and size-judgment tasks, participants were presented with a set of 64 pictures. Trial order within the block was randomized. During the picture-naming task, participants were required to name pictures depicting concrete objects in their L1. Instead, in the size-judgment task participants performed a size-judgment task on the objects depicted in the pictures (“Is this object bigger/smaller than a shoebox?”; see Dobbins et al. 2004; Branzi et al. 2016; Branzi et al. 2021). In this two-alternative forced choice setting, participants provided a yes/no response via button press. Note that participants were told that to perform the size-judgment task correctly they had to consider the size of the object in the real world. Despite the two tasks differed substantially in terms of the type of response given, they both required access to some semantic knowledge. Participants were not familiarized with the picture names beforehand to avoid repetition priming effects (Guo et al. 2011; Misra et al. 2012).

The stimuli were presented using Presentation software (Neurobehavioral systems: http://www.neurobs.com/http://www.neurobs.com/). Vocal response latencies (picture-naming task) and button press responses (size-judgment task) were recorded from the onset of the stimuli. In both tasks, each trial began with a blank screen for 1000 ms, the picture appeared for 1500 ms at the center of the screen on a black background. Then, a fixation cross was presented for 500 ms (Fig. 1).

### Behavioral data and statistical analyses

The experiment used a full within-subject design. The mean correct response rates (i.e. accuracy) and the mean reaction times of the correct trials comprised between mean reaction times ± two SD ranges were computed in the four conditions, i.e. picture-naming non-cognate (NamingNC), picture-naming cognate (NamingC), size-judgment non-cognate (Size-judgmentNC), and size-judgment cognate (Size-judgmentC), separately for each participant. To compare the cognate effect across the two tasks, we used a repeated-measures analysis of variance (ANOVA) with the within-subjects factors Task (picture-naming versus size-judgment) and Cognate Status (non-cognate versus cognate). Statistically significant interactions were assessed via planned post hoc *t*-tests. For the pairwise comparisons, we also provided an effect size value (Cohen’s *d*) and a Bayes factor value (BF10 > 3 suggests substantial evidence for a difference between the pairs and BF10 < 0.3 suggests substantial evidence for a null effect, see Jeffreys 1961). Reporting Bayes factors is useful for hypothesis testing because they provide a coherent approach to determining whether non-significant results support the null hypothesis over a theory, or whether the data are just insensitive.

### E‌EG recording and preprocessing

Electrophysiological data were recorded (Brain Vision Recorder 1.05; Brain Products) from 38 TiN electrodes placed according to the 10–20 convention system. An electrode placed on the tip of the nose was used as a reference. Two bipolar electrodes were placed next to and above the right eye to register ocular movements. Electrode impedances were kept below 5 kΩ, and EEG signal was recorded with a high cut-off filter of 200 Hz, with a sampling rate of 500 Hz. Offline EEG pre-processing involved EEG data being pre-processed offline using Fieldtrip (Oostenveld et al. 2011). Continuous EEG signals were bandpass filtered (standard non-causal two-pass Butterworth filters) between 0.1 and 100 Hz and bandstop filtered (48–52 and 98–102 Hz) to remove line noise at 50 and 100 Hz. Data were epoched from 1000 ms before the stimulus onset to 1000 ms after stimulus onset. Epochs from preprocessed data were zero-padded in the pre-stimulus (i.e. from −5000 to −1000 ms) and post-stimulus windows (i.e. from +1000 to 5000 ms) before performing time-frequency analysis. Trials and channels with artifacts were excluded by visual inspection before applying an independent component analysis (ICA) to remove components related to ocular artifacts. Excluded channels were then interpolated using the method of triangulation of nearest. After re-referencing the data to an average reference across all electrodes, the remaining trials with artifacts were manually rejected by a final visual inspection (on average, 6.77 (SD = 4.37) trials per participant, i.e. 5.30 (SD = 3.41) % of total trials per participant).

### E‌EG data processing and statistical analyses

Only the correct trials were included in the statistical analysis. Time-frequency decomposition was conducted to each electrode using a Morlet wavelet (width: 5 cycles, from 1 to 40 Hz; 1 Hz step and 20 ms time steps) and frequency analyses were performed for each trial in the four conditions, i.e. NamingNC, NamingC, Size-judgmentNC, and Size-judgmentC, separately for each participant. Furthermore, background fractal activity was attenuated in the time-frequency representation (TFR) by subtracting 1/f characteristic from the spectral power using an iterative linear fitting procedure (Griffiths et al. 2021). This step generated two vectors: one vector contained the values of each wavelet frequency A, while the other vector contained the power spectrum for each electrode-sample pair B. Both vectors were then put into log-space to approximate a linear function to get the slope and intercept of the 1/f curve. The linear equation Ax = B was resolved using least-squares regression, where x is an unknown constant describing the curvature of the 1/f characteristic. The 1/f fit Ax was then subtracted from the log-transformed power spectrum B. The corrected power was then averaged across trials in separate conditions for each participant.

The differences in power between the two contrasts NamingNC versus NamingC and Size-judgmentNC versus Size-judgmentC were first statistically assessed by applying dependent samples *t*-tests using Monte-Carlo cluster-based permutation tests (Maris and Oostenveld 2007) with an alpha cluster-forming threshold set at 0.05, three minimum neighbor channels, 2000 iterations, and cluster selection based on maximum size. Cluster-based permutation statistics were performed on the mean beta power (25–35 Hz) averaged across the time-windows of interest determined in the contrasts NamingNC versus NamingC and Size-judgmentNC versus Size-judgmentC. This step allowed us to determine two regions of interest: the region of interest in the picture-naming task contained the electrodes showing a significant difference in beta power between NamingNC versus NamingC. The region of interest in the size-judgment task contained the electrodes showing a significant difference in beta power between Size-judgmentNC versus Size-judgmentC. Furthermore, the normalized beta power relative to a baseline preceding the stimulus onset (−700 to −200 ms with respect to stimulus onset) was averaged across the significant electrodes of the regions of interests in the 25–35 Hz frequency band for the four conditions separately and exported for statistical assessments. First, the mean of normalized beta power was compared against zero in the four conditions by applying one-sample *t*-tests (two-tailed) to confirm a significant decrease in beta activity during stimulus processing. The *P*-values were corrected for multiple comparisons using the Bonferroni correction (α = 0.0125). Second, the differences in beta power between conditions were statistically assessed by means of a repeated-measures ANOVA with Task (picture-naming versus size-judgment) and Cognate Status (non-cognate versus cognate) as within-subjects factors. Statistically significant interactions were further assessed via planned post hoc *t*-tests. For the pairwise comparisons we also provided an effect size value (Cohen’s *d*) and a Bayes factor value (Jeffreys 1961). The same procedure (i.e. extraction of normalized power and statistical analyses) was applied for the analysis of 3–7 Hz theta and 8–12 Hz alpha bands. To anticipate the results, we did not find any significant effect of alpha activity during picture-object processing, across all conditions (see Supplementary Fig. S1). Thus, the remaining planned analyses were restricted to beta and theta frequencies.

We performed a searchlight-based analysis to contrast the amplitude of the cognate effect on beta power in the cluster of interest against all the remaining electrodes of the scalp. This approach allowed us to infer the extent to which the cognate effect observed in the regions of interest effectively deviated from the rest of the scalp. First, we iterated through each electrode randomly, excluding all the electrodes from the original region of interest. This constraint allowed comparing the size of the cognate effect from the original region of interest against everywhere else over the scalp. Second, we identified its immediate neighbors to create a mini-cluster for each iteration, i.e. nearest electrodes to the iteration electrode in the two-dimensional space. For each iteration, the size of the mini cluster was of 11 neighbor electrodes in the picture-naming task, and of 8 neighbor electrodes in the size-judgment task. The size of the mini-clusters (11 or 8 electrodes) matches the size of the significant clusters revealed by the cluster-based analyses performed on the difference in beta power between non-cognate versus cognate conditions, respectively, in the picture-naming and size-judgment tasks. Third, we computed the mean amplitude of the beta power (25–35 Hz) in the non-cognate and cognate conditions within this mini-cluster, in the time-window of interest determined by the previous analysis, respectively, [160–260 ms] for the picture-naming and [260–380 ms] for the size-judgment task. Following this step, the mean value was contrasted between the non-cognate and cognate conditions for each mini-cluster, and the resulting *t-*statistic was added to a distribution describing the amplitude of the cognate effect on beta power across the scalp, for the picture-naming and size-judgment tasks, separately. Finally, the *P*-value was derived by comparing the cognate effect in the regions of interest to the scalp distribution with a permutation test (2000 permutations).

### Theta-beta phase-amplitude coupling (PAC) and statistical analyses

As reported above, in this analysis, we did not consider the alpha frequency band because, we did not find any significant effect of alpha activity during picture-object processing, across all the conditions (see Supplementary Fig. S1). Instead, we investigated whether theta-beta coupling would reflect a mechanism for the collaborative functioning of lexical-semantic activation (beta effect) and response monitoring (theta effect) during response selection. PAC analyses were performed on the time-windows and regions of interest determined in the previous EEG analysis step. The two regions of interest corresponded to the electrodes where the cluster-based analysis revealed a significant difference in beta power between non-cognate and cognate stimuli in the picture-naming and in the size-judgment tasks. We expected the strength of theta-beta coupling to be modulated by an increase in control demands for cognates during the picture-naming task versus the size-judgment task.

To assess the extent to which the beta activity coupled with the theta phase, we calculated the modulation index (MI) from the correct trials only (Tort et al. 2010; Griffiths et al. 2021; Biau et al. 2022). First, the peaks in the theta and beta frequency bands were calculated by estimating power across all electrodes from the regions of interest in the picture-naming task (NamingNC and NamingC) and the size-judgment task (Size-judgmentNC and Size-judgmentC), with the same time-frequency decomposition method as above. The most prominent peaks in the theta (3–7 Hz) and beta (13–30 Hz) bands measured during picture-naming and size-judgment tasks were extracted for each participant using the Matlab function “findpeaks.” In the picture-naming task, the mean theta peak across participants was found at 5.04, SD = 0.13 Hz and the mean beta peak was found at 29.84, SD = 0.79 Hz. In the size-judgment task, the mean theta peak across participants was found at 5, SD = 0.13 Hz and the mean beta peak was found at 29.59, SD = 0.65 Hz. To obtain an equal number of trials across conditions before the MI calculation, the same number of trials across all conditions was determined by taking 80% of the smallest number of available correct trials across all the conditions (average minimum number of trials: 22.61, SD = 3.59). The 80% subsampling was done to ensure that some participants were not overrepresented in the resampling procedure due to using 100% of their available data, as well as to vary the set of trials in the condition determining the minimum number of trials across iterations (Keitel et al. 2018). Second, the time-series of the electrodes of interest were duplicated and filtered separately: the first time-series was filtered around the theta peak (SD = 0.5 Hz) and the second time-series was filtered around the beta peak (SD = 5 Hz). Then, the Hilbert transform was applied to the theta and beta filtered time-series to extract the phase of the former and the power of the latter. Then, beta power was binned into 12 equidistant bins of 30° according to the theta phase. The binning was computed for each trial and electrode separately. The MI was computed by comparing the observed distribution to a uniform distribution for each trial and electrode. The MI was then averaged across the trials and electrodes in each condition separately for statistical assessments. That is, first the mean PAC was compared against zero in the four conditions by applying one-sample *t*-tests (two-tailed) to confirm that beta activity coupled to theta phase during visual object processing. The *P*-values were corrected for multiple comparisons using the Bonferroni correction (α = 0.0125). Then, the statistical differences in mean PAC across conditions were assessed via a repeated-measures ANOVA with Task (picture-naming versus size-judgment) and Cognate Status (non-cognate versus cognate) as two within-subject factors. Statistically significant interactions were further assessed via planned post hoc *t*-tests.

*Data and scripts availability statement.* Data and scripts to reproduce the results reported in this manuscript will be made available upon publication of the manuscript. Further information or requests should be directed to the corresponding authors.

## Results

### Behavioral results

**Fig. 2:**
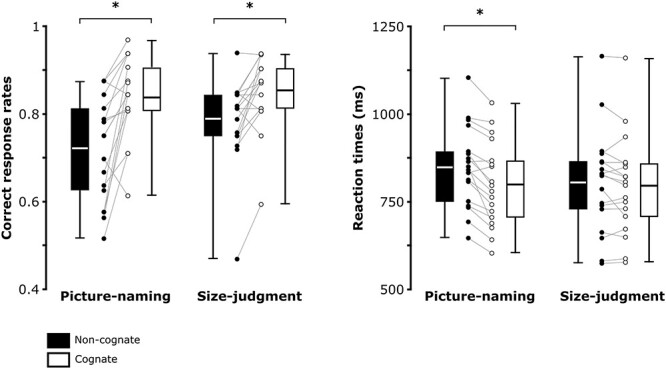
Behavioral results. Boxplots reflect accuracy (proportion of correct responses) and reaction times (ms) in the picture-naming and size-judgment tasks. Significant differences are evidenced with black stars. The bottom part of the boxplots indicates the first quarter of the score distributions, the upper part of the boxplots indicates the third quarter of the score distributions. The error bars indicate the minimum and maximum scores from the distributions.

The effect of “intention to name an object” on lexical access was assessed by comparing the cognate effect across the two tasks. The results relative to mean accuracy measures (proportion of correct responses) are reported in Fig. 2: NamingNC: 0.72, SD = 0.12; NamingC: 0.84, SD = 0.09; Size-judgmentNC: 0.79, SD = 0.10 and Size-judgmentC: 0.85, SD = 0.09. The ANOVA’s results revealed a significant main effect of Cognate Status, suggesting that retrieving cognates is easier than retrieving non-cognates [*F* (1,17) = 30.22, *P* < 0.001, η*p2* = 0.64]. Although a tendency towards significance was observed, the main effect of Task [*F* (1,17) = 3.12, *P* = 0.09, η*p*2 = 0.16] and the interaction between Task and Cognate Status were not significant [*F* (1,17) = 3.52, *P* = 0.08, η*p*2 = 0.17].

**Fig. 3:**
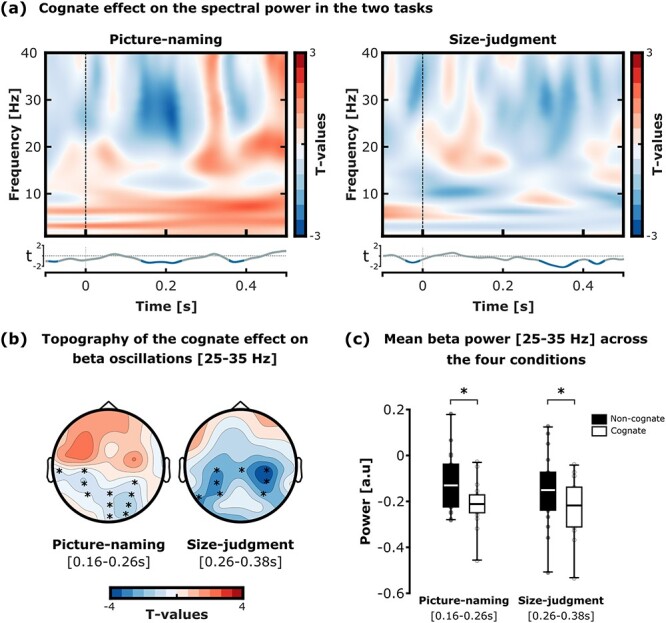
Cognate effect modulation on beta power (25–35 Hz) during the picture-naming and size-judgment tasks. (a) TFRs of the spectral power difference, i.e. cognate versus non-cognate, in the picture-naming (left) and the size-judgment task (right). The TFRs depict the difference in power for cognate versus non-cognate rather than the opposite contrast to follow conventional color codes and facilitate visualization (i.e. red indicates increased synchronization for cognate as compared to non-cognate and blue indicates increased desynchronization for cognate as compared to non-cognate). The TFRs depict the average of all electrodes included in the significant clusters, evidenced with black stars in the topography plots of panel b. the line below the two TFRs represents the *t*-values from the statistical comparisons of the mean 25–35 Hz beta power between the cognate versus non-cognate condition for each time-point (the time-points with a significant difference are evidenced in blue; *P*-values were cluster-corrected for multiple comparisons). (b) Topographies of the difference in beta power between the cognate versus non-cognate condition in the picture-naming (left) and size-judgment task (right). The time-windows of interest for the picture-naming and size-judgment task were, respectively, 160–260 and 260–380 ms after the stimulus presentation onset. The electrodes included in the significant clusters are evidenced with black stars. (c) Boxplots of the mean beta power (25–35 Hz) across the cognate and non-cognate conditions and tasks (significant differences are evidenced with black stars). The time-windows of interest for the picture-naming and size-judgment task were, respectively, 160–260 and 260–380 ms after the stimulus presentation onset. The bottom part of the boxplots indicates the first quarter of the score distributions, the upper part of the boxplots indicates the third quarter of the score distributions. The error bars indicate the minimum and maximum scores from the distributions.

The mean reaction times across conditions are depicted in Fig. 2: NamingNC: 848, SD = 115 ms; NamingC: 799, SD = 120 ms; Size-judgmentNC: 805, SD = 147 ms and Size-judgmentC: 795, SD = 142 ms. In line with accuracy results, results revealed a significant main effect of Cognate Status, suggesting that responses for non-cognates were overall slower as compared to those for cognates [*F* (1,17) = 25.153, *P* < 0.001, η*p2* = 0.597]. The main effect of Task was not significant [*F* (1,17) = 0.97, *P* = 0.338, η*p2* = 0.054]. However, the interaction between Task and Cognate Status was significant [*F* (1,17) = 11.814, *P* = 0.003, η*p2* = 0.41]. Planned comparisons revealed that the cognate effect, i.e. cognate conditions being faster as compared to non-cognate conditions, was observed in the picture-naming task only [Naming: *t* (17) = −5.366, *P* < 0.001 (adjusted), Cohen’s *d* = −1.265, BF10 = 1020.672; Size-judgment: *t* (17) = −1.286, *P* = 0.216 (adjusted), Cohen’s *d* = −.303, BF10 = 0.869].

Finally, in a set of exploratory analyses, we further examined the relationship between the behavioral cognate effect measured in the picture-naming and the size-judgment tasks. Results from Pearson’s correlation analyses revealed no significant correlation between the cognate effects measured in the two tasks (Supplementary Fig. S5b).

To summarize, the behavioral results suggest that the “intention to name an object” is not mandatory to observe lexical/phonological access. However, the cognate effect modulated both reaction times and accuracy in the picture-naming task only. Therefore, the “intention to name an object” might modulate the strength of lexical/phonological activity (cognate effect).

### Picture-object processing and lexical access lead to modulations in beta (but not alpha) frequency band

The effect of “intention to name an object” on lexicalization processes was assessed by comparing the TFRs between non-cognate versus cognate trials in the picture-naming and size-judgment tasks. This analytic step served to determine the time-window of interest containing the cognate effect indexing lexical access through oscillatory response modulations. Firstly, the TFRs in Fig. 3a did not suggest any difference in power in the 8–12 Hz alpha band (see Supplementary Fig. S1). In contrast, the TFRs suggested a stronger decrease in power in the expected beta frequency band (25–35 Hz) when comparing cognate versus non-cognate conditions in both tasks ( Fig. 3a). In the picture-naming task, the cognate effect was observed between 160 and 260 ms after the stimulus onset. In the size-judgment task, instead, the cognate effect was observed in a later time-window, i.e. between 260 and 380 ms after stimulus onset. The cluster-based analysis in these specific time-windows confirmed a significant difference in the decrease in beta power (25–35 Hz) in the cognate as compared to the non-cognate conditions, in both the picture-naming [time-window: 160–260 ms; P < 0.001, t-sum (17)  = 26.94] and the size-judgment task [time-window: 260–380 ms; P < 0.001, t-sum(17) = 18.44]. No significant negative cluster was found. Figure 3b depicts the topographies relative to beta desynchronization for the cognate versus non-cognate contrast for the two tasks separately. These results reveal that the cognate effect is reflected by beta desynchronization in similar regions of the scalp (centro-parietal scalp region) in the two tasks.

We further examined whether picture-object processing induced significant beta desynchronization across all conditions (see Piai and Zheng 2019). First, the mean normalized beta power was computed for all conditions (correct trials only) across the significant electrodes and in the time-windows of interest (Fig. 3). The results for the picture-naming task (significant electrodes: T3, C3, CP3, CP4, P3, P4, Pz, POz, PO2, Oz, and O2; time-window: 160–260 ms) and the size-judgment task (significant electrodes: T5, Cz, C3, C4, CP3, CP4, P3, and P4; time-window: 260–380 ms) are reported in Fig. 3c. Then, four one-sample *t*-tests revealed a significant decrease in mean beta power in response to the stimulus presentation across all conditions [NamingNC: -0.13, SD = 0.13, *t* (17) = −4.278, *P* < 0.001, Cohen’s *d* = −1.008, BF10 = 67.324; NamingC: -0.21, SD = 0.1, *t* (17) = −9.318, *P* < 0.001, Cohen’s *d* = −2.196, BF10 = 318.049; Size-judgmentNC: -0.15, SD = 0.16, *t* (17) = −4.01, *P* < 0.001, Cohen’s *d* = −.945, BF10 = 40.68; Size-judgmentC: -0.22, SD = 0.13, *t* (17) = −7.346, *P* < 0.001, Cohen’s *d* = −1.731, BF10 = 15785.3; *P*-values were considered significant under α = 0.0125 for multiple comparison correction].

In line with our hypothesis, these results confirm that picture-object processing induced significant beta desynchronization independently from the task and condition. The ANOVA’s result revealed a significant effect of Cognate Status [*F* (1,17) = 12.56, *P* = 0.002, η*p2* = 0.43], establishing a greater beta desynchronization for cognates as compared to non-cognates. However, no significant effect of Task [*F* (1,17) = 0.1, *P* = 0.75, η*p*2 < 0.001] or interaction between Task and Cognate Status [*F* (1,17) = 0.14, *P* = 0.71, η*p*2 < 0.001] was found. Thus, these results confirmed a greater beta desynchronization when participants processed cognate as compared to non-cognate conditions, independently from the task. Furthermore, the searchlight-based analysis revealed that the magnitude of the cognate effect on beta power was significantly greater in the regions of interest than in any other size-matching searchlight-based regions over the scalp (*P* < 0.001 in both the picture-naming and the size-judgment tasks; see Supplementary Fig. S3 for histogram of searchlight statistics). To control for potential confounds driven by the difference in the electrode pool sizes, we performed the same time-frequency decomposition of the spectral power difference with a common pool of electrodes, overlapping the two clusters of interest reported in the picture-naming and size-judgment tasks. For this control analysis, the common cluster of interest contained the following electrodes: C3, CP3, P3, C4, CP4, P4, Cz, CPz, and Pz. Results replicated the present results, with the processing of picture-objects inducing a significant beta desynchronization across all tasks and conditions. Furthermore, the cognate conditions induced a greater beta desynchronization as compared to non-cognate conditions in both tasks (see Supplementary Fig. S2).

Crucially, we also verified that the timing difference relative to the cognate effect (beta desynchronization) observed between the picture-naming (160–260 ms after stimulus onset) and the size-judgment tasks (260–380 ms after stimulus onset) did not reflect only differences in the type of (verbal versus manual) response preparation processes. To this end, we assessed the difference in the power spectrum between the picture-naming and size-judgment tasks, after collapsing the cognate and non-cognate trials together (see Supplementary Fig. S5a). The resulting TFR showed that there was no difference in power in the low frequencies of interest (<35 Hz) within the time-window containing the cognate effect (i.e. from 160 to 380 ms after stimulus onset). Note that this result also suggests that any between-task difference in beta power between 160 and 380 ms is unlikely to reflect differential response preparation processes (word production versus button press response) between the two tasks.

Finally, since we found that the cognate status modulated both behavioral and beta oscillatory responses, we conducted further exploratory analyses to test whether the magnitude of the neural cognate effect (i.e. beta desynchronization) predicted the magnitude of the behavioral cognate effect (i.e. accuracy measures). We did so by conducting Pearson’s correlation analyses for the two tasks separately. Results did not reveal any significant correlation between behavioral performance and beta responses for the non-cognate versus cognate contrast (see Supplementary Fig. S4).

Together, the key results from this analysis revealed that (1) picture-object recognition and lexical/phonological activity modulated neural responses in the beta-frequency band (Fig. 3a and c) and (2) beta-band neural effects occur earlier when the task requires explicit word-retrieval, in line with previous studies (Strijkers et al. 2012). However, (3) the strength of lexical/phonological activity (magnitude of the cognate effect) reflected by beta oscillations seems to be independent of whether the task requires explicit retrieval of the object name (Fig. 3c). Finally, (4) the beta-band neural effects reflecting lexical/phonological activity showed similar scalp distribution in the two tasks, i.e. parietal and occipital electrodes, in line with previous reports (Fig. 3b) (Strijkers et al. 2012).

**Fig. 4:**
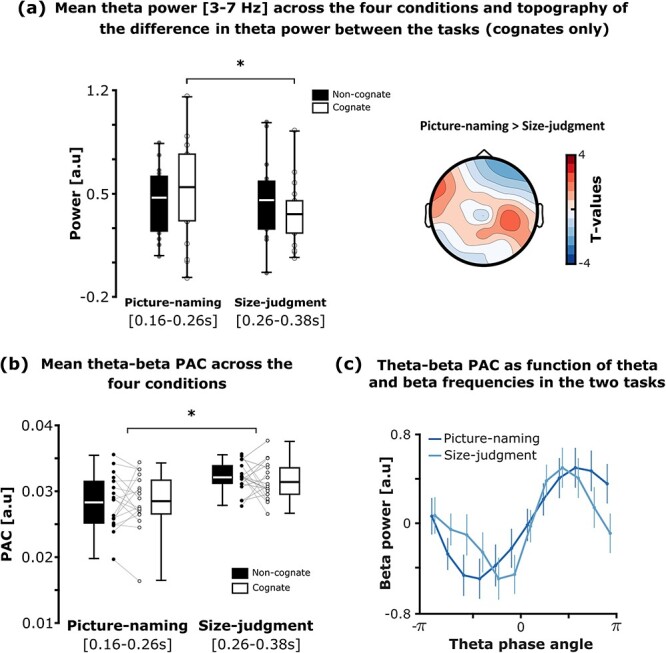
Cognate modulation on theta power (3–7 Hz) and theta-beta phase-amplitude coupling (PAC) in the picture-naming and size-judgment tasks. (a Left) boxplots of the mean theta power (3–7 Hz) across the cognate and non-cognate conditions and tasks (significant difference evidenced with a black star). The time-windows of interest for the picture-naming and size-judgment task were, respectively, 160–260 and 260–380 ms after the stimulus presentation onset. The bottom part of the boxplots indicates the first quarter of the score distributions, the upper part of the boxplots indicates the third quarter of the score distributions. The error bars indicate the minimum and maximum scores from the distributions. (a Right) topography of the difference in theta power (picture-naming versus size-judgment task) in the cognate condition only. No significant cluster was found at the scalp level. (b) Boxplots of the mean theta-beta PAC across the cognate and non-cognate conditions and tasks (significant difference is evidenced with a black star). The time-windows of interest for the picture-naming and size-judgment tasks were, respectively, 160–260 and 260–380 ms after the stimulus presentation onset. The bottom part of the boxplots indicates the first quarter of the score distributions, the upper part of the boxplots indicates the third quarter of the score distributions. The error bars indicate the minimum and maximum scores from the distributions. (c) Beta power as a function of theta phase in the regions of interest, for the picture-naming and size-judgment tasks (non-cognate and cognate trials collapsed together within each task). The fluctuation of beta power (*y*-axis) during the size-judgment task appears to concentrate more towards the theta phase (0° on the *x*-axis) as compared to the picture-naming task. Although this last analysis provides visual support only, results suggest that more narrow-band oscillations account for theta-beta PAC in the size-judgment task as compared to the picture-naming task.

### Picture-object processing and increased monitoring demands during picture-naming modulate theta activity

We examined whether picture-object processing induced a significant increase in theta power reflecting monitoring processes during response selection, independently from the condition or the task (for a review, see Piai and Zheng 2019). We applied this analysis directly in the two time-windows of interest determined above (i.e. exhibiting the greater cognate effect on beta oscillation responses in the two tasks). In line with our hypothesis, we found that theta activity (3–7 Hz) increased during picture-object processing as compared to pre-stimulus baseline (Fig. 4a, left panel). Four one-sample *t*-tests confirmed a significant increase in mean theta activity during picture-object processing across all conditions and tasks [NamingNC: 0.47, SD = 0.23, *t* (17) = 8.93, *P* = < 0.001, Cohen’s *d* = 2.104, BF10 = 181214.807; NamingC: 0.55, SD = 0.34, *t* (17) = 6.84, *P* < 0.001, Cohen’s *d* = 1.613, BF10 = 6869.73; Size-judgmentNC: 0.46, SD = 0.27, *t* (17) = 7.19, *P* < 0.001, Cohen’s *d* = 1.695, BF10 = 12298.681; Size-judgmentC: 0.36, SD = 0.22, *t* (17) = 6.95, *P* < 0.001, Cohen’s *d* = 1.639, BF10 = 8271.203; *P*-values were considered significant under α = 0.0125 for multiple comparison correction]. A repeated-measures ANOVA with Task (picture-naming versus size-judgment) and Cognate Status (non-cognate versus cognate) as within-subject factors revealed a significant interaction [*F* (1,17) = 5.609, *P = 0.0*3, η*p2* = 0.248]. Planned pairwise comparisons revealed an increase in theta power in the picture-naming task versus the size-judgment task for cognates [*t* (17) = 2.341, *P* = 0.032 (adjusted), Cohen’s *d* = 0.552, BF10 = 4.048], but not for non-cognates [*t* (17) = 0.272, *P* = 0.788 (adjusted), Cohen’s *d* = 0.064, BF10 = 0.302). The main effect of Task [*F* (1,17) = 2.677, *P* = 0.12, η*p2* = 0.136] and Cognate Status [*F* (1,17) = 0.094, *P* = 0.762, η*p2* = 0.006] were not significant. Although not significant, the topoplot reflecting the theta power difference between the picture-naming versus the size-judgment task for the cognate condition only (Fig. 4a, right panel) overlaps with the topographies reported for the cognate effect reflected by beta desynchronization (Fig. 3b).

Together, these results confirmed that picture-object processing induces an increase in theta activity across all conditions. Interestingly, and in line with our hypothesis, the results revealed an increase in theta activity for cognate conditions in the picture-naming task versus the size-judgment task. The same effect was not observed for non-cognates.

### Picture-object processing and the type of task modulate theta-beta PAC

Once established a significant increase in theta across all conditions, we investigated whether the activation of knowledge (i.e. semantic, lexical, and phonological) indexed by beta activity interacted with processes supported by theta activity (i.e. monitoring control) during lexical access. Thus, using cross-frequency coupling analysis, we evaluated the functional relationship between theta phase and beta amplitude during picture-object processing in the two tasks. In detail, we determined the participant-specific peaks in theta and beta power from the regions and time-windows of interest identified in the previous analyses, and we used the MI (Tort et al. 2010; Biau et al. 2022) to approximate theta-beta PAC (Fig. 4b and c).

First, we probed whether theta-beta coupling was indeed observed during picture-object processing, across all conditions and tasks. To do so, we tested the mean PAC values against zero in the two time-windows of interest, previously determined for the picture-naming and size-judgment tasks, where the cognate effect on beta oscillatory responses was observed (Fig. 4b). Four one-sample *t*-tests revealed a significant mean theta-beta PAC during stimulus processing, across all conditions [NamingNC: 0.028, SD = 0.004, *t* (17) = 32.23, *P* < 0.001, Cohen’s *d* = 7.597, BF10 = 3.350e+13; NamingC: 0.029, SD = 0.004, *t* (17) = 29.493, *P* < 0.001, Cohen’s *d* = 6.951, BF10 = 8.285e+12; Size-judgmentNC: 0.032, SD = 0.002, *t* (17) = 60.06, *P* < 0.001, Cohen’s *d* = 14.156, BF10 = 6.354e+17; Size-judgmentC: 0.031, SD = 0.003, *t* (17) = 48.259, *P* < 0.001, Cohen’s *d* = 11.375, BF10 = 1.951e+16; *P*-values were considered significant under α = 0.0125 for multiple comparison correction]. The ANOVA’s results revealed a significant effect of Task on theta-beta PAC [*F* (1,17) = 20.345, *P* < 0.001, η*p2* = 0.545]. Instead, the main effect of Cognate Status [*F* (1,17) = 0.009, *P* = 0.925, η*p2* = 0.0005] and the interaction between Task and Cognate Status [*F* (1,17) = 2.064, *P = 0.1*69, η*p*2 = 0.108] were not significant. Finally, as no cognate effect on theta-beta PAC was observed in either the picture-naming or the size-judgment task, non-cognate and cognate trials were collapsed together to further explore the difference in theta-beta coupling between the tasks. Results revealed that beta power’s fluctuation concentrated more towards the theta phase during the size-judgment task as compared to the picture-naming task (Fig. 4c).

To summarize, despite theta-beta interactions were triggered across all conditions and tasks, these interactions were overall stronger during the size-judgment task as compared to the picture-naming task. Interestingly, the cognate status did not modulate the strength of the interaction between theta and beta frequencies.

## Discussion

Recent research work has shown that during object recognition tasks the intention to name an object modulates neural responses reflecting activation of words (Strijkers et al. 2012; Branzi et al. 2021). However, previous literature has not clarified yet various aspects regarding the nature of this modulation. What are the neural mechanisms underpinning lexicalization processes? Is lexical access achieved differently depending on the task at hand? The present study answers these questions with some key results, as summarized below.

### Lexical access is enabled by beta desynchronization independently from the task

First, we established that lexicalization processes occur even in absence of explicit word-retrieval for oral production. In fact, behavioral and neural cognate effects were observed not only during the picture-naming task, but also during the size-judgment task. Similar results have been observed also by Strijkers et al. (2012), who manipulated word frequency and examined whether ERP responses time-locked to picture-object’s presentation varied depending on whether the task required explicit word-retrieval (picture-naming task) or whether it required a semantic judgment, but not explicit word-retrieval (semantic categorization task). Their results revealed a lexical frequency effect, irrespective of the intention to name an object. Nevertheless, the interpretation of this effect as being purely lexical was limited by the fact that word frequency tends to correlate with visual and conceptual variables. In other words, it is unclear whether the frequency effect observed by Strijkers et al. (2012) was purely lexical, or rather reflected activation of a combination of visual, conceptual, and lexical information. In the present study, we manipulated the cognate status of the stimuli, which is not correlated with any perceptual or conceptual variable (Costa et al. 2000; Costa et al. 2005; Christoffels et al. 2007; Strijkers et al. 2010; Palomar-Garcia et al. 2015). Therefore, we can confidently conclude that lexicalization processes indexed by the cognate effect occur irrespective of the intention to name an object.

Interestingly, the present results contrast with our recent functional magnetic resonance imaging study (Branzi et al. 2021) (but see results in Supplementary Fig. S6). Indeed, we found that explicit word-retrieval in the picture-naming task facilitated activation of lexical/phonological representations (measured via cognate effect), by modulating functional connectivity between areas involved in visual object recognition and phonological control. However, this neural cognate effect was not observed in the size-judgment task, which did not require explicit word-retrieval of the object names. The apparent discrepancy between our previous results and the present study might be due to the type of bilinguals tested. In the present study, bilinguals were early and high-proficient in both languages. In our previous study, instead, multilinguals were much less proficient in their non-native third language (i.e. L3) as compared to their L1. Language proficiency might explain these inconsistencies because the cognate effect, which measures the languages’ co-activation, is modulated by the strength of the links between conceptual and lexical/phonological representations (Brenders et al. 2011). Therefore, it is possible that in our previous study (Branzi et al. 2021), we did not observe a neural cognate effect in the size-judgment task because the semantic analyses required to perform this task were too superficial to engage the weak links between concepts and L3 lexical/phonological representations.

The results reviewed above suggest that lexicalization processes are triggered by visual picture-object processing, independently of the intention to name an object, and the strength of the links between conceptual and lexical/phonological representations might determine the extent to which spreading activation from the semantic system reaches the lexical and phonological representations.

Our findings indicate that the intention to name an object “speeds up” lexical access mechanisms as compared to a non-verbal context. Indeed, picture-object presentation and the cognate effect induced beta desynchronization at similar centro-parietal scalp regions in both tasks, suggesting that lexical access in the brain is likely supported by the very same neural mechanism. However, the timing of such cognate-related beta desynchronization differed depending on the task and took place earlier in the picture-naming task (160–260 ms) as compared to the size-judgment task (260–380 ms).

Interestingly, Strijkers et al. (2012) reported similar time-windows for the neural effect reflecting lexical access in a verbal versus non-verbal task comparison. In their study, the ERPs elicited by naming objects with low-frequency names started to diverge from those with high-frequency names as early as 152 ms after stimulus onset. Instead, during non-verbal categorization, the same frequency effect appeared 200 ms later (~350 ms after stimulus onset). However, regarding the nature of the modulation induced by the intention to speak, the authors reached a conclusion different from ours. Based on the observation that the ERPs measured in these two different time-windows elicited two qualitatively different neural responses in the two tasks (i.e. a P2 and a N400), the authors concluded that lexical access was relying on qualitatively different neural processes. That is, not only the two tasks differed because of the speed with which concepts triggered word activation, but also in the way words were activated.

A plausible alternative to explain the qualitative differences in the ERPs across tasks might reside in differences in the experimental paradigms. In fact, in Strijkers et al. (2012), the semantic categorization task did not differ from the picture-naming task only because it did not require explicit word-retrieval. A key difference was that the semantic categorization task was a Go/No-go task, and therefore, in the semantic categorization task, the analyzed trials were no-go trials. Differently from those measured in the picture-naming task (go trials), no-go trials require to withhold a response, a cognitive operation that typically elicits modulation of the N2 and N400 components, rather than the P2. This interpretation is in line with the finding of a N400 word frequency effect for no-go trials (Strijkers et al. 2012). Consequently, it remains unclear whether the different ERP components observed in the two tasks by Strijkers et al. (2012) reflected different types of lexicalization processes, differences in the experimental paradigm employed, or a combination of both.

In contrast, our study involved two tasks requiring a response for each trial and manipulated a variable that reflects a pure lexical/phonological effect. Our results indicate that lexical/phonological access during object naming and object categorization likely origins from the same spreading activation mechanism, and that the intention to speak speeds up lexical access enabled by power decreases in beta oscillations (Piai and Zheng 2019; Amoruso et al. 2021; Geng et al. 2022).

Finally, despite not being the focus of the present study, we analyzed the ERP correlates of the cognate effect in both tasks, to allow further comparison with previous ERP findings ( Strijkers et al. 2012). In the picture-naming task, the expected ERP cognate effect was observed in the early time-window (160–260 ms). However, no significant ERP cognate effect was observed in the size-judgment task (see Supplementary Fig. S6). These results highlight the importance of measuring lexical access effects in the frequency domain.

### Monitoring control during lexical access is reflected by theta synchronization

A second key result refers to the finding that theta (3–7 Hz) increased during picture-object processing, across all conditions and tasks. As for the responses observed in the beta band, the fact that theta activity was significantly modulated by picture-object processing in the two tasks, suggests that theta oscillations may support similar cognitive operations in verbal and non-verbal semantic tasks, although with different timings (earlier in the picture-naming as compared to the size-judgment task). This interpretation aligns with previous evidence that established an association between theta rhythms and retrieval of information across different domains, including language and memory (Jensen and Lisman 1998; Bastiaansen et al. 2005; Lisman and Buzsaki 2008). In the language domain, previous studies have reported 4–8 Hz theta power increases and 8–25 Hz alpha–beta power decreases in association with the retrieval of lexical-semantic information from long-term memory (Bastiaansen et al. 2005; Piai et al. 2014b; Piai et al. 2015; Piai et al. 2016; Piai et al. 2017; Geng et al. 2022). Furthermore, various electrophysiological studies have employed interference paradigms to investigate the control processes deployed to solve lexical/semantic competition during the picture-name retrieval and showed a link between these processes and theta activity (Piai et al. 2014a; Shitova et al. 2017; Krott et al. 2019). The view that theta oscillations synchronize when conflict between representations increases (Cavanagh and Frank 2014; Cohen 2014; Geng et al. 2022), accords with our finding of increased theta power during the picture-naming task versus size-judgment task for cognates only. In fact, if the intention to name an object induces strong dual-language activation, this should affect especially cognates, because only this category of stimuli receives direct activation from both languages. This dual-language activation should increase conflict between target and non-target cognate words. Since during picture-naming only one of the two languages (the target language) must be selected, the processing of cognates as compared to non-cognates may require greater monitoring control to avoid erroneous responses (selection of the non-target language). We propose that such increase in monitoring control is reflected by the cognate-related increase in theta activity observed in the picture-naming task as compared to the size-judgment task.

Noteworthily, although dual-language activation occurs also in the size-judgment task, as indicated by the significant cognate effect, such effect seems not only to occur earlier, but also to be stronger in the picture-naming task. In fact, in the latter, the processing of cognate versus non-cognate stimuli resulted in increased accuracy as well as faster performance. However, in the size-judgment task, the processing of cognate stimuli as compared to non-cognate stimuli  increased accuracy only . Therefore, the greater synchronization of theta oscillations observed for cognates during the picture-naming task versus the size-judgment task likely reflects the consequences of stronger dual-language activation induced by the intention to name an object. Despite resulting in facilitatory behavioral performance for cognate stimuli, dual-language activation might have increased the need of monitoring control processes to ensure that the target language is selected (Li and Gollan 2018).

### The task modulates theta-beta PAC during picture-object processing

We investigated whether the functional relationship between theta and beta activities depended on the intention to name an object and the cognate status of the stimuli by means of PAC analysis. Our results revealed, first, that the coupling at the regions of interest was greater in the size-judgment as compared to the picture-naming task. Second, in both tasks the cognate status did not influence the cooperation between the two oscillatory activities.

The present study may be the first to directly compare theta-beta PAC between a verbal and a non-verbal semantic task, using the same stimuli. If the theta-beta coupling reflects a mechanism for the collaborative functioning of lexical activation (beta effects) and response monitoring (theta effects), although speculative, a plausible interpretation is that the increase in theta-beta coupling observed for the size-judgment task might reflect increased monitoring control during the activation of object-related lexical/semantic information (beta activity). In fact, since lexical representations get activated even without the intention to speak, additional control processes might be required to ensure that the correct response (“yes” or “no” response in the size-judgment task) is produced as output, rather than the object name itself. Future studies are needed to establish if control demands during language tasks affect neural coupling between theta-beta frequencies.

## Conclusion

The present study provides new evidence for the psycholinguistic models of language production and beyond. The presence of a cognate effect in both our tasks contrasts with concept selection models, which postulate that activation will propagate from concepts to lexical and phonological representations only under the intention to name an object (Levelt 1989; Bloem and La Heij 2003). Instead, models incorporating spreading activation from the semantic to the lexical system appear to better account for these data. Accordingly, any activated conceptual representation would lead to activation of lexical information, no matter what the goal of the task is ( Dell 1986; Caramazza 1997; Levelt et al. 1999). To account for the different time course of the cognate effect between the two tasks, the principle of spreading activation specified in the models above should take into account the importance of contextual aspects and top–down knowledge that proactively impact the interaction between concepts and lexical representations.

Furthermore, our results show that word-retrieval in verbal and non-verbal semantic tasks rely on similar oscillatory dynamics for response selection and activation of lexical information, a finding which aids the current debate on the domain-specificity of the neural processes supporting language core-operations (Fedorenko and Shain 2021; Ivanova et al. 2021). In doing so, here we demonstrate that a core language operation, such as lexical access, may occur in absence of language production. This finding has important implications for understanding the performance of both healthy individuals and neurological patients in verbal and non-verbal semantic tasks.

## Supplementary Material

R1_Branzi_et_al_SI_251122_bhac499

## Footnotes

1 L1 could be either Catalan or Spanish. Therefore, depending on the participant’s L1, the picture-naming task could be performed either in Catalan or Spanish. The fact that the picture-naming task was performed either in Catalan or Spanish should not affect the cognate effect’s results. In fact, for each participant the cognate effect (non-cognate versus cognate contrast) is an effect measured within the same language.
