# Supplementary material for: Activating words without language: beta and theta oscillations reflect lexical access and control processes during verbal and non-verbal object recognition tasks
Source: Cereb Cortex. 2023 Feb 1;33(10):6228–40. doi: 10.1093/cercor/bhac499 (PMC10183750; doi:10.1093/cercor/bhac499)
Supplement: R1_Branzi_et_al_SI_251122_bhac499 [file r1_branzi_et_al_si_251122_bhac499.docx]

Supplementary Information for

Activating words without language: Beta and theta oscillations reflect lexical access and control processes during verbal and non-verbal object recognition tasks

Francesca M. BRANZI, Clara D. MARTIN, and Emmanuel BIAU

**This Supplementary file includes:**

Figure S1

Figure S2

Figure S3

Figure S4

Figure S5

Figure S6

**Cognate effect (non-cognates *versus* cognates) modulation on alpha (8-12 Hz) oscillations during the picture-naming and size-judgment tasks.**

We addressed whether mean alpha power increased during the picture-naming and size-judgment tasks, as compared to the pre-stimulus baseline (**Figure S1**). Four independent one-sampled *t*-tests (two-tailed) revealed no significant increase (or decrease) of mean alpha activity during stimulus processing across all conditions (NamingNC: 0.06, standard deviation (SD) = 0.28, *t* (17) = 0.94, *p* = 0.36, Cohen’s *d* = 0.22; NamingC: 0.03, SD = 0.27, *t* (17) = 0.47, *p* = 0.65, Cohen’s *d* = 0.11; SemanticNC: -0.14, SD = 0.34, *t* (17) = -1.78, *p* = 0.09, Cohen’s *d* = -0.42; SemanticC: -0.19, SD = 0.33, *t* (17) = -2.47, *p* = .02, Cohen’s *d* = -0.58; *P*-values were considered significant under α = .0125 for multiple comparison correction). A two-way repeated-measures ANOVA with the factors Task (picture-naming *versus* size-judgment) and Cognate Status (non-cognate *versus* cognate) revealed a significant effect of Task on mean alpha power [F (1,17) = 11.11, *p* < .001, η*p*2 = .40]. No effect of Cognate status [F (1,17) = 1.80, *p* = .20, η*p*2 = 0.1] or interaction between Task and Cognate status [F (1,17) = 0.05, *p* = .83, η*p*2 < .001] was found.

**
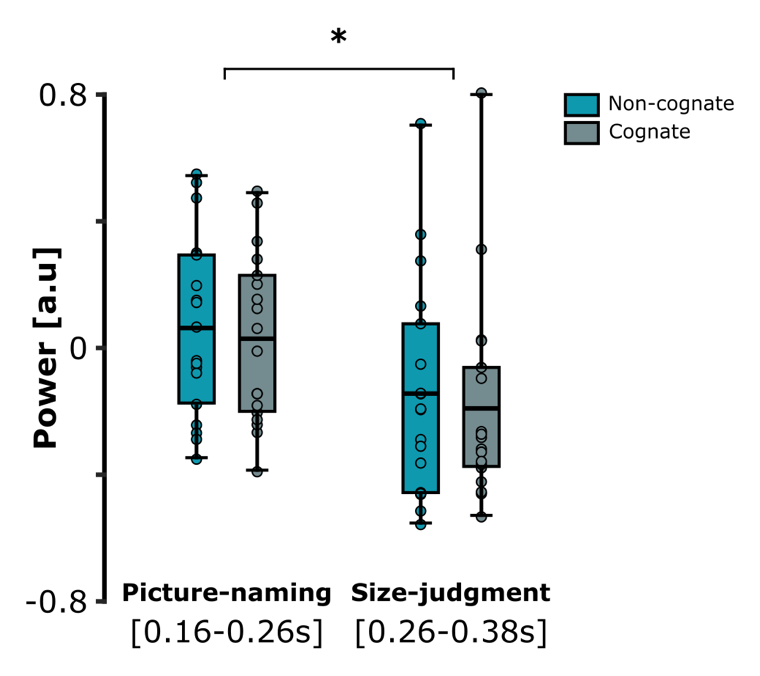
**

**Figure S1. Boxplots of the mean alpha power (8-12 Hz) for cognate and non-cognate conditions in both tasks.** The time-window of interest for the picture-naming and size-judgment tasks were respectively 160 to 260 ms and 260 to 380 ms after the onset of stimulus presentation. The significant differences between conditions are evidenced with black stars. The bottom part of the boxplots indicates the first quarter of the score distributions, the upper part of the boxplots indicates the third quarter of the score distributions. The error bars indicate the minimum and maximum scores from the distributions.

**Cognate effect in the picture-naming and size-judgment tasks with the same pool of electrodes.**

We tested whether the main result reported in the manuscript (i.e., cognate effect reflected by beta desynchronisation, occurring in distinct time-windows for the picture-naming and the size-judgment tasks), was not driven by the difference of electrode pools included in the analysis. To this end, we re-run the exact same analysis (cognate *versus* non-cognate) on the time-frequency decomposition of the spectral power difference in the two tasks, but with a common pool of overlapping electrodes. In this control analysis, for both tasks the cluster of interest contained the following electrodes: C3, CP3, P3, C4, CP4, P4, Cz, CPz, and Pz (**Figure S2**). The normalised mean beta power with respect to baseline preceding stimulus presentation was computed for all conditions across the common cluster in the two time-windows of interest (picture-naming: 160-260 ms; size-judgment: 260-380 ms with respect to the stimulus onset). The four independent one-sample *t*-tests (two-tailed) confirmed that the mean beta power was significantly below zero, across all conditions: NamingNC: -0.14, SD = 0.15, *t* (17) = -3.79, *p* = .001, Cohen’s *d* = -0.89; NamingC: -0.23, SD = 0.1, *t* (17) = -10, *p* < .001, Cohen’s *d* = -2.4; Size-judgmentNC: -0.14, SD = 0.15, *t* (17) = -3.87, *p* = .001, Cohen’s *d* = -0.91; Size-judgmentC: -0.21, SD = 0.15, *t* (17) = -6.02, *p* < .001, Cohen’s *d* = -1.42; *P*-values were considered significant under α = .0125 for multiple comparison correction). The ANOVA’s result confirmed a significant effect of Cognate status [F (1,17) = 13.67; *p* = .002, η*p2* = .45], establishing a greater beta desynchronisation for cognates as compared to non-cognates. No significant main effect of Task [F (1,17) = 0.01, *p* = .91, η*p*2 < .001] or interaction between Task and Cognate status [F (1,17) = 0.24, *p* = .63, η*p*2 = .01] was found. Therefore, picture-object processing induced a significant beta desynchronisation independently from the task and condition, in line with the results reported in the main text (**Figure 3**). Further, the cognate stimuli induced a greater beta desynchronisation as compared to non-cognate stimuli in both tasks. Altogether, this control analysis confirms that even when performed on a common cluster of overlapping electrodes between the picture-naming and size-judgment tasks, the results remain unchanged.


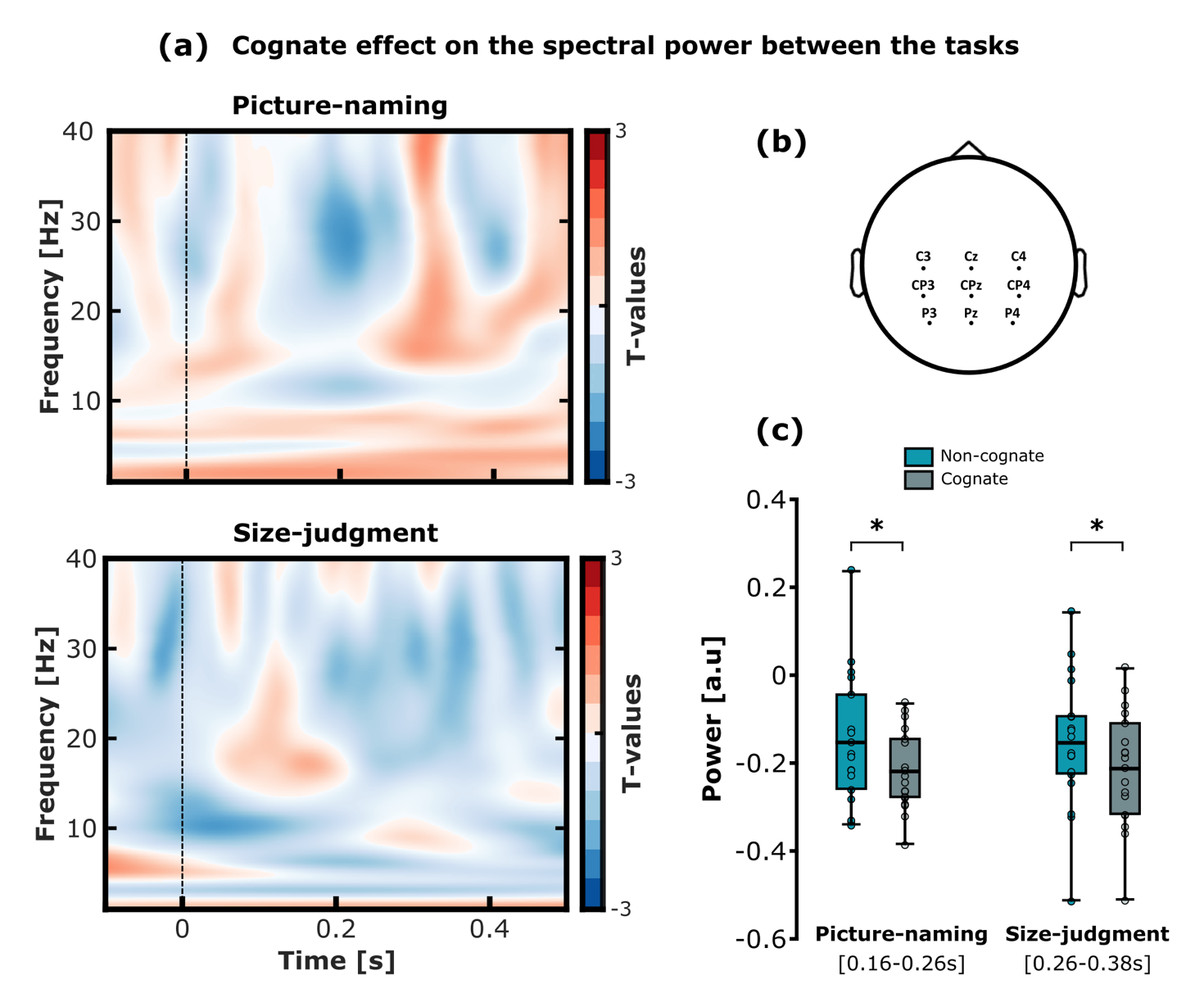


**Figure S2. Cognate effect (cognates *versus* non-cognates) modulation on beta oscillations during the picture-naming and size-judgment tasks, using the same pool of electrodes.** (a) Time-frequency representations (TFRs) of the spectral power difference, i.e., cognate *versus* non-cognate, in the picture-naming (upper panel) and in the size-judgment task (bottom panel). The TFRs depict the difference of power for cognate *versus* non-cognate rather than the opposite contrast to follow conventional colour codes and facilitate visualisation (i.e., red indicates increased synchronisation for cognate as compared to non-cognate and blue indicates increased desynchronisation for cognate as compared to non-cognate). The TFRs represents the average of all electrodes included in the common cluster depicted in the topoplot of panel b. (b) Pool of electrodes constituting the common cluster used in the control analysis: C3, CP3, P3, C4, CP4, P4, Cz, CPz, and Pz. (c) Boxplots of the mean beta power (25-35 Hz) across the cognate and non-cognate conditions and tasks (significant differences between conditions are evidenced with black stars). The time-windows of interest for the naming and size-judgment tasks were the same as those reported in the manuscript, i.e., 160 to 260 ms and 260 to 380 ms after the stimulus onset, respectively. The bottom part of the boxplots indicates the first quarter of the score distributions, the upper part of the boxplots indicates the third quarter of the score distributions. The error bars indicate the minimum and maximum scores from the distributions.

**Searchlight-based analysis assessing the magnitude of the cognate effect on beta power over the scalp.**

We performed a searchlight-based analysis to contrast the amplitude of the cognate effect on beta power (i.e., cognate *versus* non-cognate) in the significant clusters of interest against all the remaining electrodes of the scalp (**Figure S3**). This analysis revealed that the cognate effect modulation on beta power in the regions of interest was significantly greater than in any other size-matching searchlight-based regions over the scalp (*p* < .001 in both the picture-naming and size-judgment tasks).


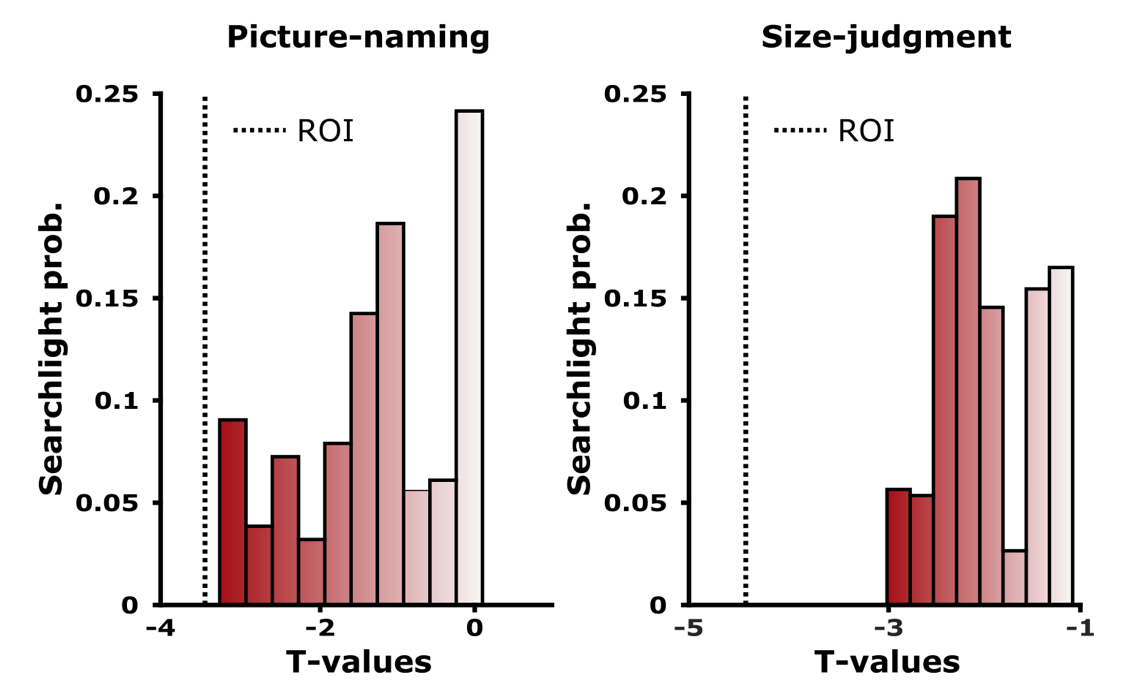


**Figure S3. Distribution of *t*-statistics for the cognate effect on beta power in the regions of interest for every searchlight over the scalp, in the two tasks.** The absolute *t*-value for the regions of interest (cognate *versus* non-cognate; *t*-values of the ROIs evidenced by the dashed line) was greater than the random searchlight-based mini-clusters matching the size of the regions of interest for both the picture-naming and size-judgment tasks (total number of permutations = 2000; *p*-values < .001).

**Correlation between neural (beta and theta power) and behavioural (accuracy) cognate effect in the picture-naming and size-judgment tasks.**

In a set of exploratory analyses, we examined whether the decrease in beta band observed for the cognate effect predicted differences in accuracy between non-cognate and cognate conditions. We correlated (Pearson’s correlation) the non-cognate *versus* cognate difference of normalized mean beta power (25-35 Hz) from the regions of interest [Δpower = beta power non-cognate - beta power cognate] with the non-cognate *versus* cognate difference of correct responses [Δcr = CRnon-cognate - CRcognate] in the picture-naming and size-judgment tasks separately (**Figure S4**). For each participant, the mean beta power (25-35 Hz) was computed in the four conditions with the same procedure reported in the manuscript. The results revealed no significant correlation between the behavioural (Δcr) and neural (Δpower) cognate effect, either in the picture-naming (*r* = 0.07, *p* = .78; two-tailed) or in the size-judgment task (*r* = 0.19, *p* = .46; two-tailed).


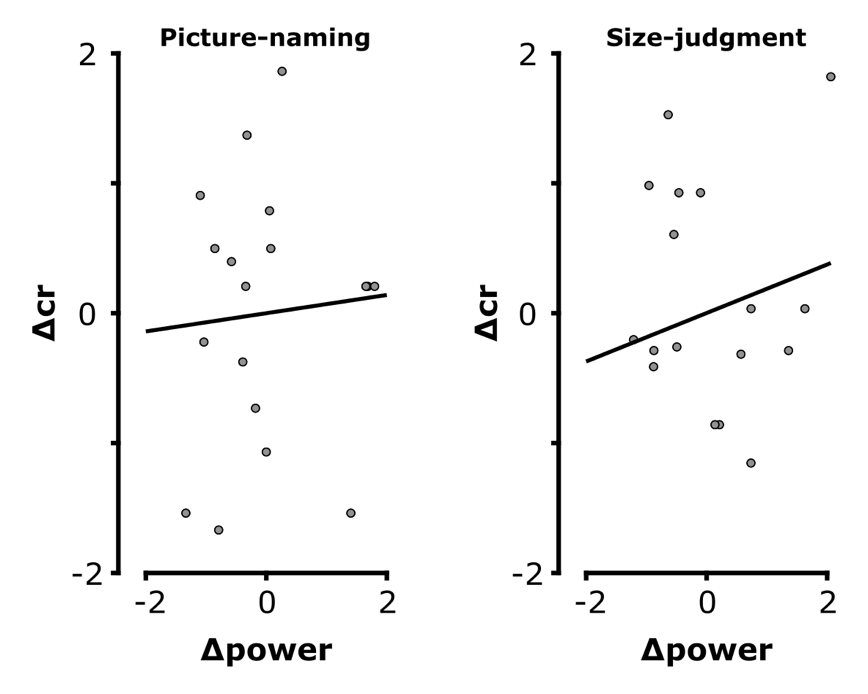


**Figure S4. Correlations between behavioural (accuracy) and neural (beta power) cognate effect measured in the picture-naming and the size-judgment tasks separately.** The difference of beta power between the non-cognate *versus* cognate condition (Δpower; *x*-axis) did not significantly correlate with the difference of accuracy between the non-cognate *versus* cognate condition (Δcr; *y*-axis) either in the picture-naming task (left scatterplot) or in the size-judgment task (right scatterplot).

We also examined whether the increase in theta band found for cognates in picture naming *versus* size judgment task predicted differences in accuracy for the same contrast. Therefore, for cognates only we correlated (Pearson’s correlation) the between-tasks difference (picture naming *versus* size-judgment task) of normalized mean theta power from the regions of interest [Δpower = theta power cognate picture naming - theta power cognate size-judgment] with the between-tasks difference of correct responses [Δcr = CR cognate picture naming – CR cognate size-judgment]. For each participant, the mean theta power (3-7 Hz) was computed in the four conditions with the same procedure reported in the manuscript. The results revealed no significant correlation between the correct response rate differences (Δcr) and the amplitude of theta power differences (Δpower) for cognate stimuli between tasks (*r* = 0.068, *p* = .7862; two-tailed).

**Picture-naming and size-judgment tasks: between-tasks spectral power differences and between -tasks correlations of behavioural performance.**

We examined whether the timing difference relative to the cognate effect (beta desynchronisation) observed between the two tasks was driven by differences in the type of (verbal *versus* manual) response preparation processes. To do so, we investigated the difference of power spectrum between the picture-naming and size-judgment tasks, by collapsing the cognate and non-cognate trials together. The resulting TFR shows that there was no difference of power in the low frequencies of interest (< 35 Hz) within the time-window where the cognate effect was found (i.e., from 160 to 380 ms with respect to the stimulus onset) (**Figure S5a**). The cluster-based permutation tests revealed a significant positive cluster (*p* = .05, cluster size = 1.08e4) and a significant negative cluster (*p* < .001, cluster size = -4.36e4). In detail, TFRs revealed a task (picture naming *versus* size-judgment) difference in the alpha-beta band (~ from 10 to 25 Hz) around 400 ms after the picture onset. Since in the picture naming task the cognate effect was observed between 160-260 ms after stimulus onset, it is unlikely that task-differences in the type of response (observed around 400 ms after stimulus onset) account for the results reported in our study.


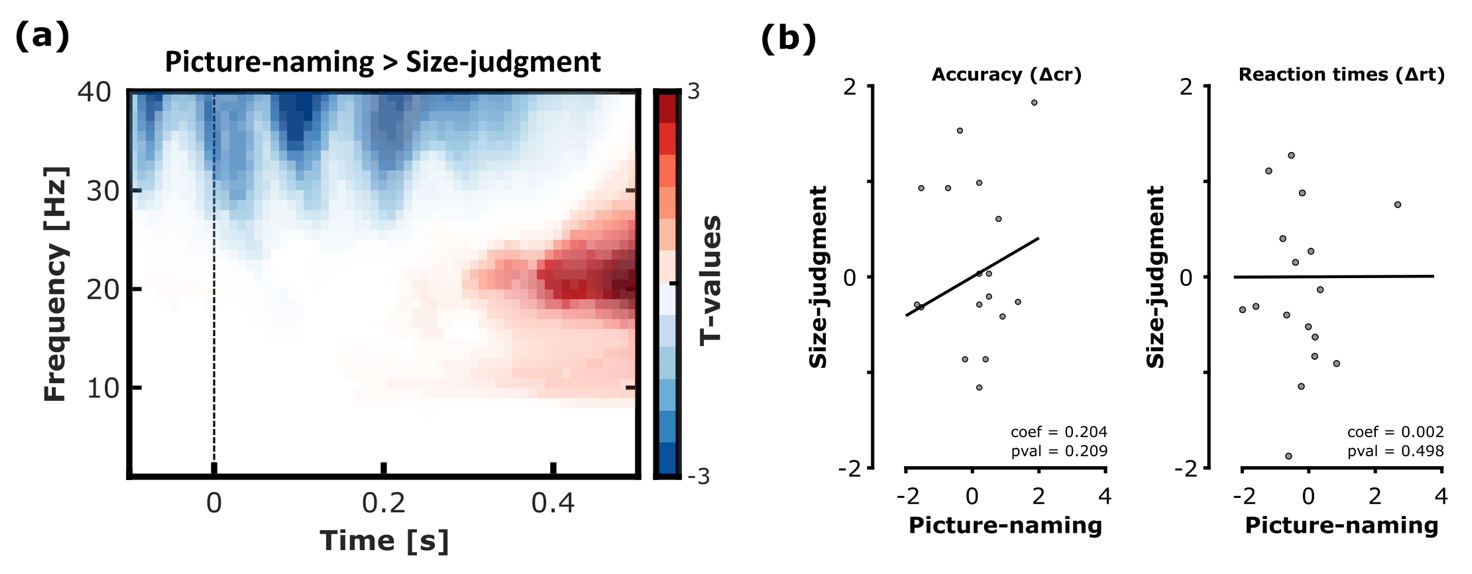


**Figure S5.** **Spectral difference of power between the picture-naming *versus* size-judgment task (and viceversa), and correlation between behavioural cognate effect in the two tasks.** (a) The TFR represents the average of electrodes included in the common cluster used in the control analysis (C3, CP3, P3, C4, CP4, P4, Cz, CPz, and Pz). (b) Left scatterplot: the difference of accuracy between the non-cognate *versus* cognate condition (Δcr) in the picture-naming task (*x*-axis) did not significantly correlate with the difference of accuracy between the non-cognate *versus* cognate condition in the size-judgment task (*y*-axis). (b) Right scatterplot: the difference of RTs between the non-cognate *versus* cognate condition (Δrt) in the picture-naming task (*x*-axis) did not significantly correlate with the difference of RTs between the non-cognate *versus* cognate condition in the size-judgment task (*y*-axis).

Finally, we examined the relationship between the behavioural cognate effect measured in the picture-naming and the size-judgment tasks. We computed the cognate effect for correct responses [Δcr = CR_non-cognate_ - CR_cognate_] as well as for RTs [Δrt = RT_non-cognate_ - RT_cognate_] in both tasks separately (**Figure S5b**). Then we correlated the behavioural cognate effects (both accuracy and RTs) between tasks, using two separate Pearson’s correlation analyses. Results revealed no significant correlation between the cognate effects in the two tasks [accuracy (Δ_CR_): *r* = 0.20; *p* = .21; two-tailed; RT (Δ_RT_): (*r* = 0.002, *p* = 0.498; two-tailed).

**Event-related potentials (ERPs) and topographies of the cognate effect in the picture-naming and size-judgment tasks.**

ERPs time-locked to the picture-object presentation were also employed to examine the cognate effect (**Figure S6**). The topography of the cognate effect was also examined in the two time-windows of interest determined by the beta oscillation analysis. For every participant, the epochs from the preprocessed data were demeaned and a baseline correction was performed in reference to pre-stimulus activity (−100 to 0 ms). ERPs were averaged for each condition and participant for further group-level analysis. ERP components were defined based on the grand averages and analysed in the time windows defined from the time-frequency analysis (i.e., 160 to 260 ms in the picture-naming task, and 260 to 380 ms in the size-judgment task). In the first window of interest (160 to 260 ms) a cluster-based analysis revealed a significant difference of amplitude between cognate *versus* non-cognate conditions for the picture-naming task (one significant negative cluster only; *p* = .022, cluster size = -10.686; two-tailed), but not for the size-judgment task (no significant positive or negative cluster; two-tailed). In the second window of interest (260 to 380 ms) the cluster-based analysis revealed no significant difference of amplitude between cognate *versus* non-cognate conditions for the picture-naming and size-judgment task (no significant positive or negative cluster).

**
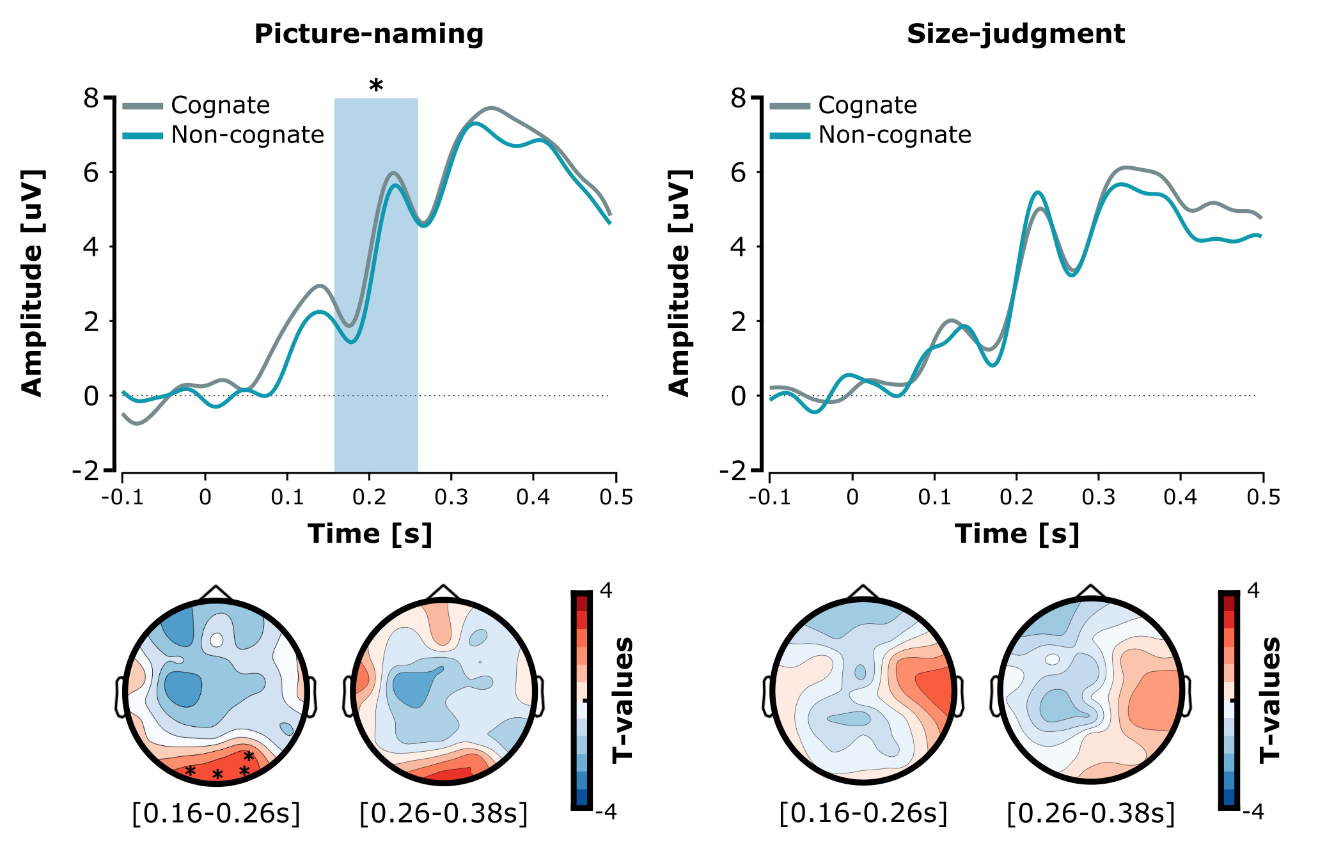
**

**Figure S6. Event-related potentials (ERPs) and topographies of the cognates *versus* non-cognates contrast in the picture-naming and size-judgment tasks.** Upper panel: Event-related potentials (ERPs) time-locked to the onset of the picture-object’s presentation in the picture-naming task (left) and size-judgment task (right). The ERP grand averages were processed across the significant electrodes revealed in the topographies below (PO2, O1, Oz and O2). The blue rectangle indicates the *a priori* time-window of interest (i.e., from 160 to 260 ms) exhibiting a significant cognate effect in the picture-naming task only. Bottom panel: Topographies reflect the cognate *versus* non-cognate ERP difference in the picture-naming (left) and size-judgment task (right). The time-windows of interest (respectively 160 to 260 ms and 260 to 380 ms after the stimulus presentation onset) were determined *a priori* from the time-frequency analyses (see Figure 3). The electrodes included in the significant clusters are evidenced with black stars (i.e., PO2, O1, Oz and O2).

In the naming task, the topography of the cognate effect on the ERPs (i.e., from 160 to 260 ms) closely overlaps with the previous beta oscillatory results reported in the centro-posterior regions (Figure S6 bottom left and see Figure 3b for visual comparison with beta response modulations). Interestingly, the difference of ERPs averaged across the significant pool of electrodes suggests that the cognate effect appears earlier as compared to its indexation with beta oscillation responses (Figure S6 left upper panel). In contrast, the ERPs were not significantly modulated by the cognate effect in the later time-window derived from time-frequency analysis on the size judgment task (i.e., from 260 to 380 ms) (Figure S6 right upper panel).
